# Apples in Amsterdam and oranges in Leiden

**DOI:** 10.1007/s12471-014-0635-8

**Published:** 2014-12-10

**Authors:** R. W. Koster

**Affiliations:** Department of Cardiology, Room G4-228, Academic Medical Center, Meibergdreef 9, 1105 AZ Amsterdam, the Netherlands

In this journal, Bosch and colleagues report their experience with out-of-hospital cardiac arrest (OHCA) in the Leiden region and report a very high survival rate to discharge of 43 % [1]. They analyse the factors that contributed to this high survival rate. Also, they note that this survival rate in the Leiden region is approximately three times higher than the European average.

The factors that seem to explain these very favourable results were the high proportion of witnessed arrests and of patients with a shockable rhythm, mostly ventricular fibrillation, some ventricular tachycardia. A high proportion of patients were initially treated with an automated external defibrillator (AED) and, last but not least, an ‘optimised chain of survival’ and the regional function of Leiden University Hospital are believed to be important for survival. Part of this optimisation is the in-hospital treatment where acute percutaneous coronary intervention (PCI) played a prominent role.

There are indeed wide variations in outcome after OHCA, and there is no full understanding of the reasons for these differences. Large variation in general medical care, education, culture, and competing illnesses may play a role in a worldwide comparison but even within one country with, generally speaking, equal health care facilities, large differences are observed [2, 3]. Understanding these differences is currently a topic of great interest [4].

For a meaningful comparison, uniform and standardised data collection and reporting is of paramount importance. The Utstein reporting methodology has been accepted worldwide since its introduction in 1991. It was revised in 2004 and a second revision is just completed [5]. The most important and absolutely critical element in reporting is adoption of a uniform initial moment in time to start the body count after onset of the OHCA. Not all persons who have a sudden death will be subjected to a resuscitation attempt: persons not waking up in the morning, otherwise unwitnessed arrests, persons with do-not-resuscitate declarations are clear but not the only examples. It is now universally accepted that ‘counting’ starts when resuscitation action such as chest compressions and mouth-to-mouth ventilation is initiated. And even that moment is not always completely clear and uniformly defined.

When resuscitation efforts are started, they may end unsuccessfully in the field, in the emergency room or even further on during hospital admission. Fortunately, efforts may also be successful, already before transport but sometimes only after prolonged resuscitation extending into the emergency room or even the cardiac catheterisation laboratory. There are well-known factors that determine that success and if many factors are unfavourable, such as unwitnessed arrest, initially recorded rhythm not ventricular fibrillation, or no bystander cardio-pulmonary resuscitation (CPR), the attempt is likely to be terminated at home and the patient will not be transported to the hospital.

This is where the current paper falls short. The data were collected from the moment that the patient enters the emergency room (with or without ongoing resuscitation) and there is no information on the events and failures in the out-of-hospital phase. This selection bias will result in an unrealistically high survival rate and an incorrect assessment of the determinants of survival, because many failures are not accounted for. And that is the reason that apples are not oranges. There is a great need, even within the Netherlands, to have a better understanding of outcomes of OHCA because the chain of survival can be optimised on the regional and local level if the numbers are known. These improvements may include immediate recognition of the need for CPR, rapid bystander CPR, early defibrillation and optimal ambulance care and post-resuscitation care. A study recently completed in the region of Noord-Holland in the context of the Arrest studies showed that survival is determined much more by actions (or the lack of actions) and circumstances in the pre-hospital phase than in the in-hospital phase of cardiac arrest and resuscitation [6]. But that does not mean that optimising the in-hospital cardiac and intensive care phase is not important and worthwhile [7].

Only with adequate and comprehensive evaluation of the process and outcome of OHCA can improvements be made and there is a great potential for improvement. Both pre-hospital measures such as extending the use of AEDs, better logistics of rapid response by lay-rescuers but also more aggressive use of PCI after restoration of spontaneous circulation and maybe even during CPR could potentially increase survival to discharge with good neurological outcome to over 40 % in all patients in which CPR is started. Such evaluation is time consuming and requires adequate resources in manpower and money but may prove very good value for money. Adhering to strict rules of data collection as described in the Utstein methodology is necessary to make sure that apples are apples and oranges are oranges.
